# Overexpression of MIEN1 in Oral Squamous Cell Carcinoma: Correlation with Tumor Invasion and Lymph Node Metastasis 

**DOI:** 10.30476/dentjods.2025.106409.2659

**Published:** 2026-09-01

**Authors:** Mahsa Kalantari, Nima Malekhoseini, Reza Malekpour Afshar, Saba Fouladi

**Affiliations:** 1 Oral and Dental Diseases Research Center, Dept. of Oral and Maxillofacial Pathology, Kerman Dental School, Kerman University of Medical Sciences, Kerman, Iran.; 2 Postgraduate Student, Dept. of Oral and Maxillofacial Surgery, Tehran Dental School, Tehran University of Medical Sciences, Tehran, Iran.; 3 Pathology and Stem Cell Research Center, Dept. of Pathology, Kerman Medical School, Kerman University of Medical Sciences, Kerman, Iran.; 4 Dept. of Clinical Biochemistry, Afzalipoor Faculty of Medicine, Kerman University of Medical Sciences, Kerman, Iran.

**Keywords:** MIEN1 protein, Neoplasm, Squamous cell carcinoma

## Abstract

**Background:**

Oral squamous cell carcinoma (OSCC) is a malignant tumor with a high potential for local invasion and distant metastasis. Migration and invasion enhancer 1 (MIEN1) is a recently identified protein that contributes to the pathogenesis of various cancers by promoting cell migration and invasion.

**Purpose:**

This study aimed to assess the expression of MIEN1 in OSCC and to compare it with its expression in dysplastic and hyperkeratotic oral epithelium.

**Materials and Method:**

In this retrospective study, MIEN1 expression was evaluated in 79 formalin-fixed, paraffin-embedded tissue samples, including 43 cases of OSCC, 23 of oral epithelial dysplasia, and 13 of hyperkeratotic oral epithelium, using immunohistochemical staining. The association between MIEN1 expression and clinicopathological parameters including patients age, sex, lesion location, tumor size, lymph node metastasis, and tumor-node-metastasis (TNM) stages was analyzed using the chi-square test, Fisher’s exact test, and Mann-Whitney test at a significance level of *p* Value< 0.05.

**Results:**

MIEN1 overexpression was significantly higher in OSCC compared to both moderate and mild dysplastic epithelium (*p*< 0.05). None of hyperkeratotic epithelial samples exhibited positive MIEN1 expression. Additionally, MIEN1 expression was significantly associated with lymph node metastasis and TNM stage (*p*< 0.05). However, no significant correlation was found between MIEN1 expression and patient age, sex, lesion location, or tumor size (*p*> 0.05).

**Conclusion:**

Our findings indicate that MIEN1 expression is correlated with the progression of OSCC. It likely plays a crucial role in the migration and invasion of cancer cells, as well as in the metastatic spread of the disease.

## Introduction

Oral cancer is a subset of head and neck malignancies, with oral squamous cell carcinoma (OSCC) accounting for over 90% of all oral cancer cases [ 1 ]. OSCC is regarded as a fatal and debilitating neoplasm due to its aggressive local invasion, destructive nature, and potential for metastasis to distant organs [ 2-6 ]. Despite significant advancements in the diagnosis and treatment of many cancers over the past decade, the management of oral cancer remains challenging, largely because of difficulties in detecting the disease at early stages. Furthermore, the global incidence of oral cancer has increased substantially, contributing considerably to the overall cancer burden worldwide [ 7-10 ]. OSCC is a multifactorial disease influenced by a variety of genetic alterations and environmental factors; however, the molecular and cellular mechanisms underlying its pathogenesis are not yet fully elucidated [ 11 ]. Lymph node metastasis is a major determinant of poor prognosis in OSCC patients. Therefore, identifying novel genes involved in tumor cell migration and invasion is crucial to improving our understanding of the molecular pathways driving OSCC progression and metastasis [ 9 - 10 ].

Migration and invasion enhancer 1 (MIEN1), also known as C35 or C17orf37, is a novel gene located on human chromosome 17q12, positioned between the HER2 and GRB7 genes [ 12 ]. It encodes a membrane-associated protein that functions as an oncogene in various cancers and plays a critical role in promoting cancer cell migration and invasion [ 13 - 14 ]. MIEN1 is predominantly localized to the inner leaflet of the plasma membrane, where it enhances the activation of matrix metalloproteinase 9 (MMP9), urokinase plasminogen activator (uPA), vascular endothelial growth factor (VEGF), as well as other proteases and angiogenic factors regulated downstream of the nuclear factor kappa-light-chain-enhancer of activated B cells (NF-κB) signaling pathway. Furthermore, MIEN1 induces remodeling of the actin cytoskeleton by promoting the formation of filopodia, facilitating cellular migration and invasion [ 15 - 16 ]. Several studies have also demonstrated that elevated MIEN1 expression correlates with inhibition of apoptosis, increased lymph node metastasis, and higher histopathological tumor grade [ 13 - 14 , 17 ].

Although numerous studies have demonstrated the involvement of MIEN1 in the progression of various cancers- including breast, prostate, colorectal, uterine, ovarian, pancreatic, melanoma, and bladder carcinomas [ 13-16 , 18-20 ]- to the best of our knowledge, only one study has investigated its role in OSCC progression [ 9 ]. Therefore, the present study aimed to evaluate MIEN1 expression in OSCC using immunohistochemical staining (IHC) and to compare it with its expression in dysplastic and hyperkeratotic oral epithelium. 

## Materials and Method

Samples of OSCC, dysplastic, and hyperkeratotic oral epithelium were obtained from patients referred to the Oral and Maxillofacial Pathology Department of Kerman Dental School between August 2017 and March 2022. The dysplastic and hyperkeratotic samples were clinically diagnosed as leukoplakia and biopsied to assess the presence of dysplastic changes. The inclusion criteria compromised histopathologically confirmed cases of OSCC, oral epithelial dysplasia and hyperkeratotic lesions with complete clinical and pathological records with no prior history of radiotherapy, chemotherapy, or other preoperative interventions. All histopathological diagnoses were independently confirmed by two pathologists. The exclusion criteria included patients who received any form of neoadjuvant therapy (chemotherapy, radiotherapy, or surgery) prior to biopsy, samples with incomplete clinical or pathological data, specimens with poor tissue preservation or inadequate quality for immunohistochemical analysis. The study protocol was approved by the Ethics Committee of Kerman University of Medical Sciences (approval code: IR.KMU.REC.1395.612).

Clinicopathological data- including age, gender, lesion location, tumor size, and presence of metastasis- were extracted and recorded. All OSCC cases were reviewed and staged independently by two pathologists according to the tumor-node-metastasis (TNM) classification of the International Union against Cancer. Histological grading was performed based on the World Health Organization (WHO) classification criteria [ 21 ]. 

IHC staining was performed on formalin-fixed, paraffin-embedded tissue blocks to evaluate MIEN1 expression in all samples. Serial 3-μm sections were cut, deparaffinized, rehydrated, and rinsed in distilled water. Antigen retrieval was carried out by boiling the sections in citrate buffer (pH 6.0) using a high-pressure microwave oven for 20 minutes, followed by cooling to room temperature. The slides were then washed in phosphate-buffered saline (PBS; pH 7.4) for 2 minutes. Sections were incubated overnight at 4°C with a primary rabbit polyclonal anti-MIEN1 antibody (Sigma-Aldrich, St. Gallen, Switzerland) diluted 1:500, followed by incubation with an anti-rabbit secondary antibody (Abcam, United States). The next day, slides were washed four times in PBS for 3 minutes each. Diaminobenzidine (D-AB) was used as the chromogen, and the reaction was stopped by rinsing with distilled water. As positive and negative controls, a breast cancer tissue sample and a section of normal oral mucosa were stained, respectively.

All stained slides were independently evaluated under a light microscope (BX-41, Olympus, Japan) by two qualified pathologists. The staining index was calculated by multiplying the intensity of nuclear staining by the proportion of stained cells. Staining intensity was graded as follows: 0 (unstained), 1 (yellow), 2 (orange), and 3 (brown). The proportion of stained nuclei was determined by counting 100 cells across five high-power fields (400× magnification) and scored as: 0 (≤5%), 1 (6–25%), 2 (26–50%), 3 (51–75%), and 4 (>75%). The final staining score was classified into four categories: 0–1 (negative), 2–4 (weakly positive), 5–8 (moderately positive), and 9–12 (strongly positive) [ 14 ].

Statistical analyses were performed using SPSS version 24.0 (IBM Corp., USA), using the chi-square test, Fisher’s exact test and Mann-Whitney test at a significance level of *p*< 0.05. 

## Results

A total of 79 cases were evaluated, including 43 OSCC, 23 dysplastic oral epithelium samples (12 moderate and 11 mild dysplasia), and 13 hyperkeratotic oral epithelium samples. Patients’ ages ranged from 15 to 92 years, with a mean age of 63.21±14.07 years. Gender distribution was nearly equal, comprising 40 males and 39 females. The most common lesion sites were the tongue (48. 1%), lips (22.8%), and buccal mucosa (11.4%). The majority of cases (83.5%) measured less than 2cm in diameter.

**Figure 1:**
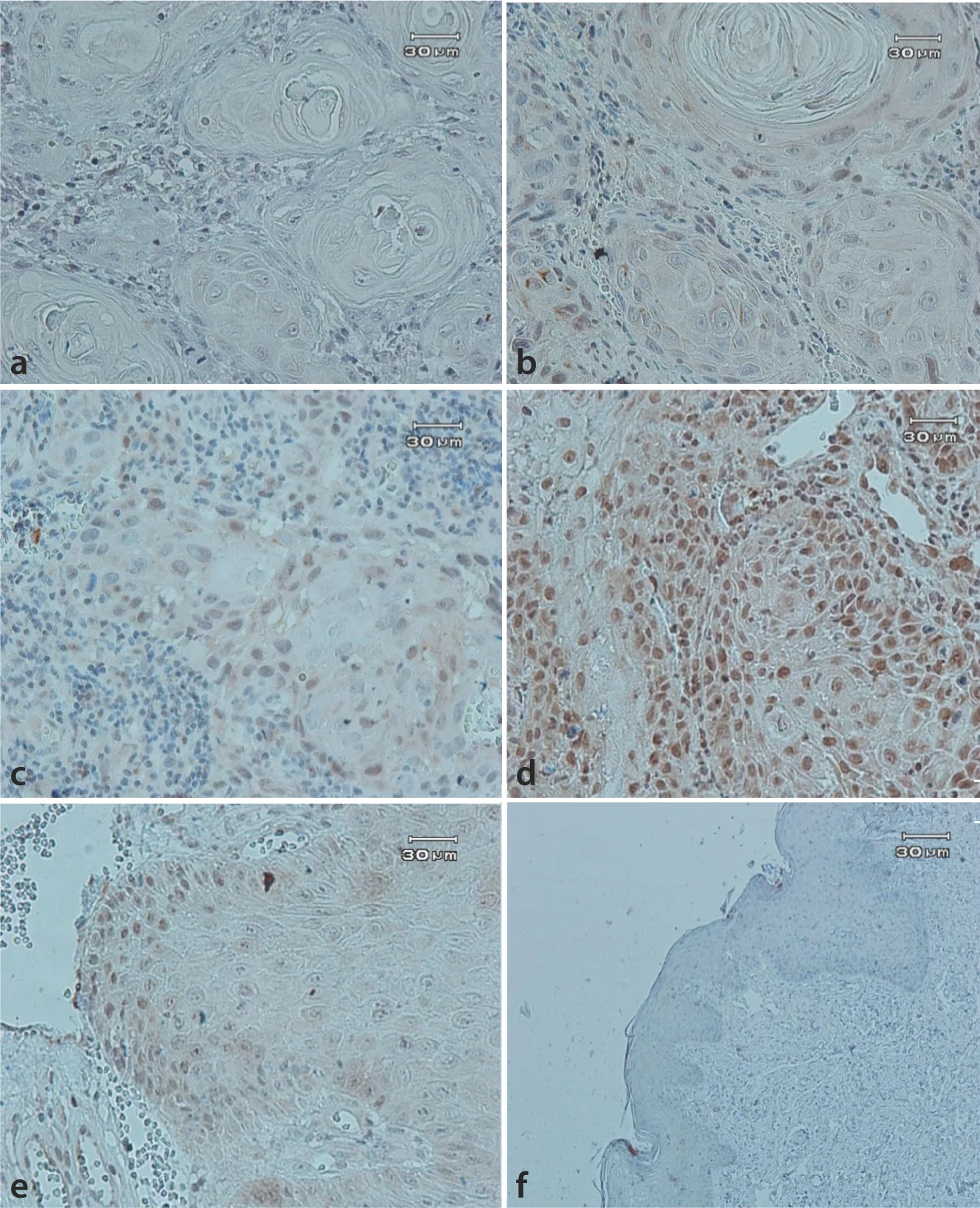
Expression of migration and invasion enhancer 1 (MIEN1) by immunohistochemical staining (400×), **a:** Negative expression in oral squamous cell carcinoma (OSCC), **b:** Weakly positive expression in OSCC, **c:** Moderately positive expression in OSCC, **d:** Strongly positive expression in OSCC, **e:** Weakly positive expression in dysplastic epithelium, **f:** Negative expression in hyperkeratotic epithelium

MIEN1 overexpression was detected in 79% of OSCC samples (34 of 43). Positive expression was observed in 83.3% of moderate dysplastic and 27.3% of mild dysplastic cases. None of the hyperkeratotic epithelial samples showed MIEN1 expression. MIEN1 was predominantly localized in the nucleus, appearing as brown or brownish-yellow granules. Most moderate dysplasia samples exhibited weakly positive MIEN1 expression (9 of 12), whereas the majority of OSCC samples showed moderate positivity (21 of 43). Only 3 of 11 mild dysplasia samples demonstrated weakly positive MIEN1 expression (Figure 1). 

MIEN1 expression was significantly higher in OSCC compared to moderate dysplasia, mild dysplasia, and hyperkeratotic epithelium, with *p* Values of 0.005, 0.05, and 0.002, respectively. No significant associations were found between MIEN1 expression and clinical parameters such as age, gender, lesion size, or location (*p*> 0.05) (Table 1).

**Table 1:** MIEN1* expression according to age, gender, location, tumor size and histopathologic diagnosis

Variable	MIEN1 expression					*p* Value
	Negative	Weakly positive	Moderately positive	Strongly positive	Total N (%)	
Age						
≤ 60	18(22.9%)	10(12.6%)	9(11.4%)	2(2.5%)	39(49.4%)	0.387
˃ 60	14(17.7%)	8(10.1%)	13(16.4%)	5(6.3%)	40(50.6%)	
Gender						
Male	17(21.5%)	7(8.9%)	13(16.4%)	3(3.8%)	40(50.6%)	0.402
Female	15(18.9%)	11(13.9%)	9(11.3%)	4(5.1%)	39(49.4%)	
Location						
Tongue	15(18.9%)	11(13.9%)	10(12.6%)	2(2.5%)	38(48.1%)	0.490
Lips	9(11.4%)	4(5.1%)	5(6.3%)	0(0%)	18(22.8%)	
Buccal mucosa	3(3.8%)	0(0%)	4(5.1%)	2(2.5%)	9(11.4%)	
Floor of the mouth	3(3.8%)	2(2.5%)	1(1.3%)	2(2.5%)	8(10.1%)	
Palate	1(1.3%)	1(1.3%)	1(1.3%)	0(0%)	3(3.8%)	
Alveolar ridge	1(1.3%)	0(0%)	0(0%)	1(1.3%)	2(2.5%)	
Vestibule	0(0%)	0(0%)	1(1.3%)	0(0%)	1 (1.3%)	
Size						
≤ 2cm	27(34.1%)	18(22.8%)	17(21.5%)	4(5.1%)	66(83.5%)	0.16
2-4cm	5(6.3%)	0(0%)	3(3.8%)	2(2.5%)	10(12.6%)	
>4 cm	0(0%)	0(0%)	2 (2.5%)	1 (1.3%)	3 (4.8%)	
Histopathologic diagnosis						
SCC	9(11.4%)	6(7.6%)	21(26.6%)	7(8.9%)	43(54.4%)	0.001*
Moderate dysplasia	2(2.5%)	9(11.4%)	1(1.3%)	0(0%)	12(15.1%)	
Mild dysplasia	8(10.1%)	3(3.8%)	0(0%)	0(0%)	11(13.9%)	
Hyperkeratotic epithelium	13(16.4%)	0(0%)	0(0%)	0(0%)	13(16.4%)	

*Migration and invasion enhancer 1 (MIEN1)

Subgroup analyses within each histopathologic diagnosis also showed no significant correlation between MIEN1 expression and age, gender, lesion size, or location in OSCC, moderate dysplasia, or mild dysplasia cases (*p*> 0.05).

Of the 43 OSCC cases, staging according to the TNM system classified 26 samples as stage I, 8 as stage II, 7 as stage III, and 2 as stage IV. All OSCC samples were histologically well differentiated. Regional lymph node metastasis was present in 34 cases, while no distant metastasis was detected. MIEN1 expression was significantly associated with the presence of regional lymph node metastasis and tumor stage (Table 2). Furthermore, MIEN1 expression levels were significantly higher in tumors classified as TNM stages III and IV compared to stage I tumors (*p*= 0.003 and 0.005, respectively). 

**Table 2:** MIEN1* expression in OSCC** samples according to TNM staging and regional lymph node metastasis

Variable	MIEN1 expression					*p* Value
	Negative	Weakly positive	Moderately positive	Strongly positive	Total N (%)	
TNM staging						
I	7 (16.3%)	6 (13.9%)	13 (30.3%)	0 (0%)	26 (60.5%)	0.003*
II	2 (4.6%)	0 (0%)	4 (9.4%)	2 (4.6%)	8 (18.6%)	
III	0 (0%)	0 (0%)	4 (9.4%)	3 (6.9%)	7 (16.3%)	
IV	0 (0%)	0 (0%)	0 (0%)	2 (4.6%)	2 (4.6%)	
Regional lymph node metastasis						
Yes	0 (0%)	0 (0%)	4 (9.4%)	5 (11.5%)	9 (20.9%)	0.004*
No	9 (20.9%)	6 (14%)	17 (39.6%)	2 (4.6%)	34 (79.1%)	

*Migration and invasion enhancer 1 (MIEN1) ** Oral squamous cell carcinoma (OSCC)

## Discussion

OSCC accounts for approximately 3% of all malignancies in the United States, with an estimated 28,900 new cases diagnosed annually [ 21 ]. The overall five-year survival rate for OSCC remains relatively low, ranging between 50–60% [ 22 - 23 ]. Identifying genetic alterations that drive tumor cell migration and invasion is essential for uncovering the molecular mechanisms underlying metastasis. Moreover, proteins affected by these alterations may serve as valuable biomarkers for diagnostic, prognostic, and therapeutic purposes in various types of cancer [ 24 ].

Previous studies have shown that MIEN1 is overexpressed in multiple malignancies, including breast, colorectal, prostate, gastric, ovarian, and non-small cell lung cancers, suggesting its involvement in tumor progression and metastasis [ 13 - 15 , 24 - 27 ]. However, the role of MIEN1 in the pathogenesis of OSCC remains largely unexplored. To date, and to the best of our knowledge, only one study has investigated the association between MIEN1 expression and OSCC progression [ 9 ]. 

Our findings revealed a significantly elevated level of MIEN1 expression in OSCC compared to moderate and mild dysplastic oral epithelium. Notably, none of the hyperkeratotic epithelial samples exhibited MIEN1 expression. Among the moderate dysplasia cases, 9 out of 12 showed weakly positive expression and only one case demonstrated moderate expression. In contrast, 21 out of 43 OSCC samples exhibited moderate MIEN1 expression, while 7 cases showed strong expression. These results suggest that MIEN1 may contribute to the transformation of normal oral epithelium into dysplastic and malignant forms. In line with our findings, Rajendiran *et al* . [ 9 ] reported MIEN1 overexpression in severe dysplasia and OSCC compared to mild dysplasia and hyperplastic oral epithelium, based on an in vitro model, patient tissue analysis, and data from The Cancer Genome Atlas (TCGA).

Interestingly, unlike previous studies on other cancers, our results demonstrated nuclear and perinuclear localization of MIEN1 in OSCC. For instance, Dong *et al* . [ 14 ] reported cytoplasmic MIEN1 expression in colorectal cancer cells, and similar cytoplasmic localization was observed in breast cancer by Yin *et al* . [ 13 ]. However, Rajendiran *et al* . [ 9 ] were the first to report a nuclear/perinuclear pattern of MIEN1 expression in OSCC, consistent with our observations. They proposed that this unique pattern- distinct from other solid tumors-may be involved in specific signaling cascades that modulate OSCC progression.

In the present study, we observed a significant positive correlation between MIEN1 expression and both regional lymph node metastasis and advanced TNM stages in OSCC. These findings suggest that MIEN1 may contribute to tumor invasion and metastatic spread to lymph nodes. Similar associations have been reported in colorectal, breast, and prostate cancers, where elevated MIEN1 expression is linked to tumor progression and metastasis [ 13 - 14 , 16 ]. Zhao *et al* . [ 27 ], for example, demonstrated that breast cancer patients with positive MIEN1 expression had significantly lower survival rates. Consistently, Rajendiran *et al* . [ 9 ] proposed that MIEN1 serves as a molecular determinant of OSCC cell invasion, metastasis, and poor prognosis. MIEN1 mediates these effects primarily through activation of NF-κB–dependent target genes, such as MMP9, uPA, and VEGF, via the PI3K/Akt signaling pathway. Additionally, MIEN1 influences actin cytoskeleton remodeling by promoting filopodia formation, thereby enhancing cellular migration, invasion, and metastatic potential. Post-translational modification of MIEN1 through prenylation at its C-terminal CaaX motif is also critical for its role in cell motility and metastasis [ 15 - 16 ].

The lymphatic vessel marker D240, as shown by Agarwal *et al* . [ 28 ], reliably differentiates lymphatic from blood vessels and correlates with lymph node metastasis in OSCC. In contrast, our findings indicate that MIEN1 overexpression offers a molecular insight into metastatic potential with comparable diagnostic accuracy. Unlike D240, which primarily marks lymphangio genesis, MIEN1 may serve as an intrinsic biomarker of tumor invasiveness. Thus, MIEN1 and D240 could be used complementarily- MIEN1 for molecular characterization and D240 for anatomical confirmation of lymphatic spread.

Our results revealed no significant associations between MIEN1 expression and patient age, gender, lesion size, or location. This aligns with previous findings in other malignancies; Dong *et al* . [ 14 ] reported no correlation between MIEN1 expression and sex or tumor size in colorectal cancer, Zhao *et al* . [ 27 ] observed similar results in breast cancer, and Yin *et al* . [ 13 ] found no relationship between MIEN1 expression and age or tumor size in breast carcinoma patients.

Previous studies have highlighted MIEN1 as a potential molecular target for cancer therapy. Liang *et al* . [ 29 ] demonstrated that miR-124-5p suppresses gastric cancer cell proliferation and metastasis by targeting MIEN1. Comparable findings were reported by Li *et al* . [ 30 ] in non-small cell lung cancer and Rajendiran *et al* . [ 31 ] in prostate carcinoma, suggesting the broader relevance of MIEN1-targeted interventions across multiple cancer types. 

## Conclusion

Our findings demonstrated a significant overexpression of MIEN1 in OSCC compared to dysplastic and hyperkeratotic oral epithelium. Additionally, MIEN1 expression was positively correlated with advanced TNM stage and regional metastasis, highlighting its potential role in tumor progression. These results suggest that MIEN1 may serve as a valuable biomarker for OSCC diagnosis and prognosis, as well as a promising therapeutic target. However, further studies with larger sample sizes are warranted to better elucidate the precise role of MIEN1 in the pathogenesis of OSCC.
